# Innovative method using circulating tumor cells for prediction of the effects of induction therapy on locally advanced non-small cell lung cancer

**DOI:** 10.1186/1749-8090-8-175

**Published:** 2013-07-16

**Authors:** Shintaro Tarumi, Masashi Gotoh, Yoshitaka Kasai, Natsumi Matsuura, Masaya Okuda, Tetsuhiko Go, Shinya Ishikawa, Hiroyasu Yokomise

**Affiliations:** 1 Department of general thoracic, breast and Endocrinological surgery, Faculty of medicine Kagawa University, Kagawa, Japan

**Keywords:** Circulating tumor cells, Non-small cell lung cancer, Induction chemoradiotherapy, Surgery, CellSearch

## Abstract

**Background:**

The existence of circulating tumor cells (CTCs) in patients with lung cancer has been reported. The purpose of this study was to assess whether CTCs are predictive of the pathological effects of induction chemoradiotherapy for patients with non-small cell lung cancer.

**Methods:**

Patients who underwent induction chemoradiotherapy followed by surgery were compared with those who underwent surgery alone. Peripheral and pulmonary venous blood samples from the involved lobe were collected intraoperatively, and the number of CTCs was counted using the CellSearch™ system, an epithelial cell adhesion molecule-based immunomagnetic technique.

**Results:**

Of the 9 patients who underwent induction therapy, 4 achieved pathological CR, 4 achieved major response, and 1 achieved minor response. All patients who underwent induction therapy and surgery alone were negative for CTCs in peripheral blood. In the induction therapy group, 4 patients showing pathological CR were negative for CTCs in pulmonary venous blood (pvCTCs) and 5 showing major/minor response were positive (mean, 57.8 cells). The numbers of CTCs in patients showing major/minor response were significantly higher than those in patients showing pathological CR (*p* = 0.012, Mann–Whitney U test). All 6 patients undergoing surgery alone were positive for pvCTCs (mean, 207.5 cells), showing a significant difference from those undergoing induction therapy (*p* = 0.038).

**Conclusions:**

The existence of CTCs in pulmonary venous blood reflects pathological non-CR, and therapeutic pathological response may be predicted by pvCTC measurement.

## Background

Primary lung cancer is the leading cause of cancer death in most industrialized countries [1]. Although early stage non-small cell lung cancer (NSCLC) is curable by surgical resection [2-4], there is no established standard therapy for resectable locally advanced NSCLC (e.g., bulky N2 disease)[5]. Induction chemoradiotherapy followed by surgery for locally advanced NSCLC remains controversial, but a favorable prognosis was reported in patients who achieved pathological complete response (CR) [6]. At present, the preoperative prediction of pathological response to induction therapy is inadequate, even with diagnostic imaging modalities such as fluorine-18-fluorodeoxyglucose positron emission tomography or computed tomography (CT) [7].

The concept of circulating tumor cells (CTCs) was described by Paget in 1889 [8]. CTCs offer potential utility as a prognostic, predictive, and/or pharmacodynamic biomarker [9-11], and CTC characterization is a research area that could yield information on the mechanisms of metastasis [12]. Previous studies demonstrated that CTCs can be detected and quantitatively evaluated in patients with primary lung cancer [10,13]. Although the detection rate of CTCs was very low in the peripheral blood of lung cancer patients with no clinically detectable distant metastasis [14-16], it was higher in pulmonary venous blood [17]. Since pathological CR is defined as a condition in which no pathologically viable tumor cells remain [18], we hypothesized that CTCs are undetectable in patients achieving CR, even in pulmonary venous blood. The purpose of this study was to assess whether CTCs are predictive of pathological response to induction chemoradiotherapy. Therefore, we counted the number of CTCs in pulmonary venous blood (pvCTCs) and peripheral blood (periCTCs) and examined their relationship with pathological response.

## Methods

### Patients

This study was conducted in Kagawa University Hospital from December 2010 to January 2012. The institute review boards of Kagawa University approved the study protocol, and all patients provided written informed consent. Patients agreeing to the purpose of the study were eligible. All patients presented with a pathological diagnosis of NSCLC and underwent an evaluation of staging by imaging studies, including contrast-enhanced whole-body CT, contrast-enhanced magnetic resonance brain imaging, and integrated positron emission tomography/CT. Patients with concurrent or prior malignancy treated within the previous 5 years were excluded.

#### Induction therapy and surgery

The details of induction chemoradiotherapy have been described previously [6]. In brief, chemotherapy was performed on weeks 1 and 5, and concurrent radiation therapy with 50 Gy (2 Gy/day, 5 days/week) was conducted from week 1 to week 5. The patients received carboplatin (area under the curve 6 mg/[mL*min], 2-h intravenous infusion) and docetaxel (60 mg/m^2^, 2-h intravenous infusion) on day 1. Pulmonary resection was performed by thoracotomy. All operations were performed by one thoracic surgeon. As much as possible, pulmonary arteries were isolated and selected as the first maneuver.

#### Estimation

The pathological effects of induction therapy were evaluated according to the *General Rules for Clinical and Pathological Records of Lung Cancer*, *7*^*th*^*edition*[18]. Multiple hematoxylin- and eosin-stained sections of each tumor were reviewed by a pathologist and then re-reviewed by another pathologist. After resectioning the entire tumor and scrutinizing multiple cross-sections, this pathologist carefully calculated the percentage of nonviable tumor cells. The percentage of nonviable tumor cells was defined as the combined percentage of scar and necrosis. Pathological CR denotes that viable tumor cells do not exist in the resected specimen; a major response denotes that viable tumor cells remained in one-third of resected specimens; a minor response denotes that viable tumor cells remained in more than one-third of resected specimens; and no response denotes that no pathological changes were observed in the tumor cells of the resected specimen. The pathologists were blinded to all clinical, radiological, and surgical findings. Pathological diagnosis was defined according to the pathologists’ comments on the clinical records.

Peripheral and pulmonary venous blood samples (7.5 mL each) from the lobe involved were collected during thoracotomy for CTC enumeration. CTCs were isolated from samples using the CellSearch™ system (Veridex LLC, Raritan, NJ, USA), the technical details of which, including accuracy, precision, linearity, and reproducibility, have been described previously [13]. CTCs were defined as antibodies against epithelial cell adhesion molecule (EpCAM)-isolated intact cells showing positive staining for cytokeratin and negative staining for CD40. All CTCs were evaluated by two persons having no knowledge of the clinical status of the patients. The finding of two or more CTCs was defined as positive [13]. Correlation between the results of CTC enumeration and pathological response was analyzed. Data were compared using the Mann–Whitney U test. The level of significance was set at 0.05. Statistical analysis of the data was performed using Statistical Package for the Social Science for Windows, version 20.0.0 (IBM SPSS , Chicago, IL, USA).

## Results

Patient characteristics are summarized in Table 1. Nine patients underwent induction chemoradiotherapy and surgery (IT group; all clinical stage 3A), and 6 underwent surgery alone (SA group). Pathological CR and major and minor responses were achieved in 4, 4, and 1 patients, respectively. In the SA group, 2 patients each were at clinical stages 1A and 1B and 1 patient each was at 2A and 2B. CTC enumeration is shown in Table 2. All patients in both groups were negative for periCTCs. Five patients in the IT group were positive for pvCTCs (mean, 57.8 cells; range, 4–108). These patients showed major or minor response, while the remaining 4 patients who achieved CR were negative for pvCTCs. A significant difference in the numbers of pvCTCs was observed between major/minor and complete response cases (*p* = 0.012, Mann–Whitney U test). All 6 patients in the SA group were positive for pvCTCs (mean, 207.5 cells; range, 4–855), showing a significant intergroup difference (*p* = 0.038).

**Table 1:** Patient characteristics

	**Group IT (n = 9)**	**Group SA (n = 6)**
Age, median (range)	66 (57–75)	77 (63–90)
Gender	Male	Male
Histological cell type		
Squamous cell carcinoma	6	1
Adenocarcinoma	3	4
Large cell carcinoma	0	1
Clinical stage		
1	0	4
2	0	2
3	9	0
T1aN2	1	
T2bN2	3	
T3N1	3	
T3N2	2	
Pathological stage*		
0	4	0
1	2	4
2	0	2
3	3	0
T1aN2	1	
T1bN2	1	
T3N1	1	
Pathological effect		
Complete response	4	-
Major response	4	-
Minor response	1	-
Operative procedure		
Lobectomy**	6	6
Pneumonectomy	3	0

Abbreviations: *IT* Induction chemoradiotherapy followed by surgery, *SA* Surgery alone.

*Pathological stage means post-therapeutic stage (yp–stage) in the IT group.

**Lobectomy, including bilobectomy and lobectomy with bronchoplasty/angioplasty.

**Table 2:** CTC enumeration

**Group**	**Pt**	**c-stage**	**p-stage**	**Pathological effect**^**†**^	**periCTCs**		pvCTCs^*^	
					**Evaluation**	**Count**	**Evaluation**	**Count**
IT	#1	3A	0	Complete	Negative	0	Negative	0
IT	#2	3A	0	Complete	Negative	0	Negative	0
IT	#3	3A	0	Complete	Negative	1	Negative	0
IT	#4	3A	0	Complete	Negative	0	Negative	1
IT	#5	3A	1A	Major	Negative	0	Positive	12
IT	#6	3A	1B	Major	Negative	0	Positive	60
IT	#7	3A	3A	Major	Negative	0	Positive	4
IT	#8	3A	3A	Major	Negative	0	Positive	5
IT	#9	3A	3A	Minor	Negative	0	Positive	108
SA	#1	1A	1A	-	Negative	0	Positive	4
SA	#2	1A	1A	-	Negative	0	Positive	51
SA	#3	1B	1B	-	Negative	0	Positive	8
SA	#4	1B	1B	-	Negative	0	Positive	216
SA	#5	2A	2A	-	Negative	0	Positive	111
SA	#6	2B	2A	-	Negative	0	Positive	855

Abbreviations: *IT* Induction chemoradiotherapy followed by surgery, *SA* Surgery alone.

Pathological effect^†^; complete, viable tumor cells do not exist at all in the resected specimen; major, viable tumor cells remained in one-third of resected specimens; minor, viable tumor cells remained in more than one-third of resected specimens.

^*^Significant difference was observed between major/minor and complete response cases (p = 0.012, Mann–Whitney U test) and between the SA and IT groups (p = 0.038).

## Discussion

The present study is the first to evaluate the relationship between CTCs and the pathological effects of induction chemoradiotherapy in NSCLC patients. CTC count was found to be significantly higher in pulmonary than in peripheral blood. In patients who underwent induction chemoradiotherapy, pvCTC count was negative in the case of CR, whereas it was positive in the other cases.

While patients who achieved pathological CR by induction chemoradiotherapy plus surgery achieved favorable prognosis [5], it is difficult to evaluate pathological CR following induction therapy. If it is possible to confirm CR patients before operation, patient selection based on surgical indications will be possible and may contribute to a prognostic improvement. Usage of pvCTCs has the potential to accurately reflect the pathological effects of induction therapy, and their measurement is useful in the prediction of pathological CR. This suggests that it would be possible to conduct a prospective study of patients achieving pathological CR by induction chemoradiotherapy. The question of whether pulmonary resection is necessary in patients achieving CR may then be easier to answer, because preoperative evaluation would be possible when a pulmonary vein catheter technique (e.g., pulmonary vein isolation) is used [19].

In the IT group, the number of patients with squamous cell carcinoma was greater than that of patients with adenocarcinoma. Three patients with squamous cell carcinoma were positive for pvCTCs and 3 were negative. Two and 1 patient with adenocarcinoma were positive and negative for pvCTCs, respectively. Okumura and colleagues reported that the incidence of positive periCTCs was significantly higher in squamous cell carcinoma than in adenocarcinoma, but no correlation was observed between pvCTC count and histological type [17]. To investigate the correlation between pvCTC count and histological type, futher study is required. The cause for the difference in the number of pvCTCs and periCTCs remains unclear. As for the result, the role of destruction within the blood stream or seeding to tissue bed is considered as a hypothesis.

The major limitation of this study is that the study population was small, and greater patient numbers are desirable for further study. Further study using a large sample size is important for making definitive conclusions. Sawabata and colleagues reported that periCTCs were detected in NSCLC cases following surgical manipulation [20]. That study also raised concerns over the use of pvCTCs. However, since periCTCs and pvCTCs (in cases showing CR) obtained intraoperatively were all negative, this concern may be ruled out.

In CTC detection, clustered cells were hardly extracted from the blood using the CellSearch™ system. EpCAM-based enrichment methods such as use of the CellSearch™ system have a limitation in that EpCAM can be downregulated during epithelial–mesenchymal transition [21]. A recent study indicated that this process is attributable to metastasis [22]. Various alternate methods are currently available [23-27], and it may be necessary to verify results using other methods.

The present study has established a new role for CTC analysis in the evaluation of induction chemoradiotherapy. While sampling of pulmonary venous blood is currently not easy, the development of a new, non-invasive procedure for sampling pulmonary venous blood would enable determination of pathological CR preoperatively. We may be able to verify the likely efficacy of surgery after induction chemoradiotherapy in the treatment of locally advanced NSCLC.

## Conclusions

No pvCTCs were found in the group showing pathological CR. Although the sample size was limited, a significant difference was observed in the numbers of pvCTCs between the CR and non-CR groups. In addition, all patients who did not receive induction chemoradiotherapy were positive for pvCTCs regardless of disease stage. The pvCTC status accurately reflected pathological CR in this study, and pathological response may therefore be predictable through pvCTC measurement.

## Abbreviations

CR: Complete response; CTCs: Circulating tumor cells; pvCTCs: CTCs in pulmonary venous blood; NSCLC: Non-small cell lung cancer; CT: Computed tomography; periCTCs: CTCs in peripheral blood; EpCAM: Epithelical cell adhesion molecule; IT: Induction chemoradiotherapy followed by surgery; SA: Surgery alone.

## Competing interests

The authors declare that they have no competing interests.

## Authors’ contributions

ST participated in the design of the study and coordination, and carried out CellSearch system, and drafted the manuscript. MG participated in the design of the study and carried out CellSearch system, and helped to draft the manuscript. YK and NM participated in the design of the study and carried out CellSearch system. MO, TG and SI participated in the design of the study and performed the statistical analysis. YH conceived of the study and participated in its design. All authors read and approved the final manuscript.
